# Diagnostic Challenges of Central Nervous System Tuberculosis 

**DOI:** 10.3201/eid1409.070264

**Published:** 2008-09

**Authors:** Laura J. Christie, Ann M. Loeffler, Somayeh Honarmand, Jennifer M. Flood, Roger Baxter, Susan Jacobson, Rick Alexander, Carol A. Glaser

**Affiliations:** California Department of Public Health, Richmond, California, USA (L.J. Christie, S. Honarmand, J.M. Flood, C.A. Glaser); Children’s Hospital and Research Center at Oakland, Oakland, California, USA (L.J. Christie); Legacy Emanuel Children’s Hospital, Portland, Oregon, USA (A.M. Loeffler); Francis J. Curry National Tuberculosis Center, San Francisco, California, USA (A.M. Loeffler); Kaiser Oakland Medical Center, Oakland (L.J. Christie, R. Baxter); Kaiser Fremont Medical Center, Fremont, California, USA (S. Jacobson); Contra Costa County Public Health Laboratory, Martinez, California, USA (R. Alexander)

**Keywords:** Tuberculosis, meningitis, encephalitis, Mycobacterium tuberculosis, Mycobacterium bovis, central nervous system, dispatch

## Abstract

Central nervous system tuberculosis (TB) was identified in 20 cases of unexplained encephalitis referred to the California Encephalitis Project. Atypical features (encephalitic symptoms, rapid onset, age) and diagnostic challenges (insensitive cerebrospinal fluid [CSF] TB PCR result, elevated CSF glucose levels in patients with diabetes, negative result for tuberculin skin test) complicated diagnosis.

Tuberculosis (TB) of the central nervous system (CNS) is classically described as meningitis. However, altered mental status, including encephalitis, is within the spectrum of clinical manifestations. Because early treatment can dramatically improve outcomes, consideration of TB as a potential pathogen in CNS infections, including encephalitis, is vital. The California Encephalitis Project (CEP), initiated in 1998 to study the causative agents, epidemiology, and clinical features of encephalitis, has identified 20 cases of culture-confirmed tuberculous encephalitis. In most instances, TB was not initially considered to be a likely cause.

## The Study

Referrals are received by the CEP statewide from clinicians seeking diagnostic testing for immunocompetent patients, including TB PCR testing when appropriate, who meet the CEP case definition of encephalitis (*1*). Mycobacterial testing was often also conducted by the referring hospital. Inclusion criteria for this report were a positive cerebrospinal fluid (CSF) culture for *Mycobacterium tuberculosis* complex or a positive CSF TB PCR result. Clinical data were compiled from case history forms and other medical records when available. To evaluate differences among causes of encephalitis, TB patients were compared with CEP patients with cases of enterovirus and herpes simplex virus 1 (HSV-1) encephalitis. Demographic, clinical, and laboratory data were compared by using the Fisher exact test, χ^2^ test, or Kruskal-Wallis test as appropriate (statistical significance was set at α = 0.05).

From June 1998 through October 2005, a total of 1,587 patients were enrolled in the CEP; 20 patients fulfilled criteria as TB cases. Demographic and clinical information for the study population are detailed in the Appendix Table. Median age was 41 years (range 8 months to 77 years). The median time from symptom onset to first lumbar puncture was 5 days (range 0–62 days). Seventeen patients (85%) had a second lumber puncture.

In general, CSF values became more abnormal over time, with increasing leukocyte counts and protein levels and decreasing glucose levels (Appendix Table). Most patients had a CSF mononuclear cell predominance, although 4 patients (21%) had a neutrophil predominance. All patients had cranial neuroimaging, magnetic resonance imaging (18 of 20), and computed tomography (20 of 20) (Appendix Table; Table). Results of computed tomography scans were often normal (50%).

Of patients in whom the results of a recent tuberculin skin test (TST) were known, 59% (10 of 17) had a negative result (Appendix Table). Many chest radiographs (9 of 18, 50%) showed no abnormalities. Concurrent culture positive pulmonary disease was found in 4 (50%) of 8 patients tested. A history of foreign birth (53%) or foreign travel (80%) was common. When these factors were reported, 5 patients (25%) had a history of treatment for TB and 5 patients (63%) had contact with a known case of TB. Only 2 patients did not have at least 1 of these risk factors.

Of the 20 cases identified, all had a positive CSF culture for *M*. *tuberculosis* complex. Only 4 (24%) of 17 were CSF TB PCR positive and none had a positive CSF acid-fast bacilli smear (Appendix Table). All but 3 patients had pan-susceptible *M*. *tuberculosis* isolates; 2 patients had *M*. *bovis* isolates (resistant to pyrazinamide) and 1 patient had an isoniazid-resistant isolate.

When patients with TB were compared with patients with viral causes of encephalitis (Table), those with enterovirus encephalitis were significantly younger, were less likely to require intensive care, had shorter hospitalizations, had fewer abnormal results for CSF and neuroimaging, and were less likely to die (all p<0.05). Patients with HSV-1 encephalitis were more likely than those with CNS TB to be white and non-Hispanic and to have shorter hospital stays, lower CSF leukocyte and protein levels, and higher CSF glucose levels.

**Table:** Comparison of TB encephalitis with viral encephalitis, California Encephalitis Project*

Characteristic	CNS TB	HSV-1	Enterovirus
Total no. cases	20	39	44
Patient demographics			
Male, no. (%)	12 (60)	14 (36)	25 (57)
Median age, y (range)	41 (8 mo–77y)	55 (8 mo–89 y)	13 (6 mo–74 y)†
Race white, non-Hispanic, no. (%)	3 (15)	22 (69)‡	14 (33)
Clinical data			
Interval from CNS onset to admission, d, median (range)	5 (0–62)	2 (0–28)	2 (0–18)†
ICU care, no (%)	15 (75)	19 (56)	20 (53)
Fever, no. (%)	15 (75)	35 (92)	35 (80)
Seizures, no. (%)	7 (35)	22 (58)	12 (27)
Altered consciousness, no. (%)	13 (65)	30 (79)	20 (50)
Personality change, no. (%)	9 (45)	17 (45)	7 (17)†
Hallucinations, no. (%)	3 (16)	7 (21)	3 (7)
Stiff neck, no. (%)	14 (70)	12 (32)§	19 (44)
Ataxia, no. (%)	7 (37)	6 (23)	11 (28)
Length of hospital stay, d, median (range)	30 (8–753)	15 (0–738)§	6 (0–1,124)¶
Laboratory results			
CSF leukocytes, per mL, median (range)	201 (42–2,845)	47 (0–975)‡	85 (0–1,080)†
CSF protein, mg/dL, median (range)	174 (66–357)	71 (15–297)‡	60 (19–881)¶
CSF glucose, mg/dL, median (range)	35 (9–132)	69 (39–112)‡	67 (38–159)¶
MRI/CT (abnormal, initial study), no. (%)	17 (85)	36 (95)	12 (39)¶
Inpatient deaths, no. (%)	6 (30)	8 (21)	4 (9)†

*CNS, central nervous system; TB, tuberculosis (*Mycobacterium tuberculosis*); HSV-1, herpes simplex virus 1; ICU, intensive care unit; CSF, cerebrospinal fluid; MRI, magnetic resonance imaging; CT, computed tomography. Denominators may vary slightly depending on available data. Data were analyzed (2-way analysis) by using Fisher exact test, χ^2^ test, or Kruskal-Wallis test as appropriate, with statistical significance set at α = 0.05. Comparisons without a symbol did not reach statistical significance. †CNS TB vs. enterovirus, p<0.05. ‡CNS TB vs. HSV-1, p<0.001. §CNS TB vs. HSV-1, p<0.05. ¶CNS TB vs. enterovirus, p<0.001.

## Conclusions

Although tuberculous meningitis is well described, prominent encephalitic features are less commonly reported. Illness and death associated with neurotuberculosis are highly dependent on the stage of disease at diagnosis; early diagnosis and treatment correlates with better outcomes (*2*). Although the TB cases reported here represent only a small percentage of CEP cases (<1%), CNS TB with an encephalitic picture warrants further discussion because of high morbidity and mortality rates and need for early diagnosis and appropriate treatment.

This study found atypical features of CNS disease that may have confounded early diagnosis. Tuberculous CNS disease is typically described as a chronic meningitis with insidious onset in children <5 years of age or in older adults with relatively few cases during school age years or adolescence (*3*). In contrast, CEP TB patients came to a hospital within 2 weeks of symptom onset and the greatest percentage of CEP TB patients was found in persons 10–19 years of age (22%).

Although typical CSF studies (mononuclear cell pleocytosis, low glucose levels, and elevated protein levels) (*4*) were often found in CEP CNS TB patients, atypical findings were noted. CSF glucose levels were often normal in patients with diabetes, although the ratio of CSF to serum glucose was invariably low. Additionally, a CSF neutrophil predominance was found in 4 patients, erroneously suggesting pyogenic meningitis. Although clinicians may be tempted to ascribe abnormal CSF values to viral meningitis or encephalitis based on abnormal CSF values, CEP patients with enterovirus and HSV-1 encephalitis rarely had glucose levels <40 mg/dL. Median protein levels were significantly higher in patients with CNS tuberculosis (174 mg/dL in TB) than in patients infected with HSV-1 (71 mg/dL) or enterovirus (60 mg/dL) (p<0.001).

Diagnostically, the low sensitivity of CSF TB PCR is problematic. Potential explanations for the lack of sensitivity in CSF specimens include low bacillary load in CSF, small sample volumes, and PCR inhibitors in the sample (*5*). Given that all of our patients had positive CSF cultures, we would have expected a higher PCR yield. Most concerning was the finding that many providers caring for these patients were dissuaded from pursuing TB as a diagnostic possibility when the PCR result was negative.

Given the difficulties in obtaining a rapid diagnosis, therapy must often be initiated empirically. Unfortunately, a history of TB and TST or chest radiograph results were not reliable indicators of active disease and might be difficult to obtain. Further complicating therapy, lengthy cultures, and isolate sensitivities are necessary to optimize choices of antimicrobial drugs. Ten percent (2) of our patients were infected with *M*. *bovis*, a relatively higher percentage than in other reports (*6*–*8*). Intrinsic pyrazinamide resistance (*9*) of *M*. *bovis* required modification of use of empiric antimicrobial drugs.

A limitation of this series is inclusion of only patients with a positive CSF culture. Because historical data suggest that only 25%–70% of patients with a diagnosis of CNS TB have a diagnosis confirmed by microbiologic testing (*2*), there were likely additional CEP patients with CNS tuberculous disease without a positive acid-fast bacilli culture who were not included in this series. Additionally, the series was limited by the referral bias inherent in the project; CEP patients are typically sicker and present greater diagnostic challenges. Thus, those with obvious or mild CNS tuberculous disease would be underrepresented. Despite this potential bias, outcomes were similar (mortality rate 30%) compared with reported mortality rates (18%–72%) and morbidity rates (16%–48%) in previous studies (*10*).

This report emphasizes some atypical features of CNS TB manifested as encephalitis. Encephalopathic changes, a relatively rapid course, nonclassic age distribution, and negative TB PCR and TST results should not dissuade a clinician from considering TB, particularly when CSF:serum glucose ratio (*11*) is <0.5 and CSF protein level is >100 mg/dL. The CEP TB patients reported here may represent a severe part of the continuum of TB meningitis or may represent a distinct encephalitic subset with atypical features.

## Supplementary Material

Appendix Table Clinical, laboratory, and radiologic results for 20 patients with tuberculous encephalitis, California Encephalitis Project*
