# Supplementary material for: Diagnostic Challenges of Central Nervous System Tuberculosis
Source: Emerg Infect Dis. 2008 Sep;14(9):1473–5. doi: 10.3201/eid1409.070264 (PMC2603083; doi:10.3201/eid1409.070264)
Supplement: Appendix Table — Clinical, laboratory, and radiologic results for 20 patients with tuberculous encephalitis, California Encephalitis Project* [file 07-0264_appT-s1.pdf]

Appendix Table. Clinical, laboratory, and radiologic results for 20 patients with tuberculous encephalitis, California Encephalitis Project*

Patient age, sex	Race	Interval from onset to admission, d	LP no. 1 CSF leukocytes (differential), protein, glucose†	LP no. 2 CSF leukocytes (differential), protein, glucose‡	TST result	CSF AFB smear	CSF AFB culture	CSF TB PCR	AFB/TB testing of respiratory specimens	Initial CXR results	Cranial MRI findings	Outcome
8 mo, F	A	1	42 (ND) 66, 35	6 (94L, 6M) 120, 37	+	–	+	–	Gastric aspirate: AFB smear –, culture –	Right middle lobe infiltrate versus adenopathy	MAS, I, H, BE	Died
5 y, F	H	0	75 (4N, 90L, 5M) 86,52	116 (46N, 42L, 13M) 131, 62	–	–	+	U		Normal	MAS, I, H, BE	Home in good condition
9 y, M	A	11	345 (9N, 88L, 3M) 155, 39	276 (7N, 92L, 1M) 222, 35	–	–	+	–		Normal	Normal	Home in good condition
12 y, F	H	62	95 (12N, 82L, 6M) 139, 36	360 (70N, 22L) 192, 40	–	–	+	U	Specimen type unknown: + *(Mycobacterium bovis)*	Normal	MAS	Discharged with mild left 6th and 7th cranial nerve palsy and severe language impairment
14 y, M	H	5	115 (10N, 90M) 92, 25	271 (52N, 44L, 4M) 92, 19	+ (1993, 1994)	–	+	–	Bronchoscopy: AFB culture +, AFB smear –, TB PCR+ (M. *bovis*)	Bilateral apical fine interstitial infiltrates and small left upper lobe granuloma initially, developed right upper lobe cavitation during TB therapy	MAS, I, BE	Slow complete recovery
18 y, F	A	20	2,845 (17N, 75L, 7M, 1E) 174, 27	456 (34N, 58L, 6M) 213, 12	+ (1997)	–	+	–		Normal	MAS, I, H, BE, ME	G tube and tracheostomy, but alert and communicative
19 y, M	A	0	108 (56N, 30L, 14M) 177, 25		U	–	+	–-	Sputum: AFB culture +, AFB smear – TB PCR+	Small left pleural effusion, no infiltrate	MAS	Died
24 y, M	H	14	733 (90N, 5L, 5M) 234, 9		U	–	+	U	Sputum: AFB culture +, AFB smear –	Bilateral interstitial and vague nodular alveolar infiltrates most marked within upper lobe, possible small right pleural effusion	MAS, I	Died
29 y, F	H	3	286 (6N, 75L) 237, 8	850 (56N, 36L, 4M, 1E) 196, 18	+	–	+	–	Sputum: AFB culture –, AFB smear –	ND	MAS	Home in good condition

Patient age, sex	Race	Interval from onset to admission, d	LP no. 1 CSF leukocytes (differential), protein, glucose†	LP no. 2 CSF leukocytes (differential), protein, glucose‡	TST result	CSF AFB smear	CSF AFB culture	CSF TB PCR	AFB/TB testing of respiratory specimens	Initial CXR results	Cranial MRI findings	Outcome
37 y, F	H	7	178 (74N, 25L, 1M) 303, 23	390 (84N, 9L, 7M) 280, 18	–	–	+	–	Sputum: AFB culture +, AFB smear –	Left pleural and parenchymal opacities, scattered calcified granulomas in bilateral upper lobes	MAS, I, H	Persistent vegetative state, intubated
45 y, M§	A	17	630 (95L, 5M) 215, 132	1,030 (85L, 13M) 381, 5	–	–	+	–-		Normal	MAS, I, H, ME	Died
52 y, M	H	7	223 (33N, 66M) 128, 14	20 (19N, 81M) 87, 63	+	U	+	+		No acute infiltrates	MAS, H, BE	VP shunt, rehabilitation
54 y, M§	H	4	357 (94L, 6M) 200, 54	592 (2N, 92L, 6M) 178, 88	–	–	+	–		Linear densities in bilateral bases, right greater than left, likely old scarring	I, H	Home with neurologic deficits
59 y, M	A	21	303 (26N, 74M) 127, 33	404 (25N, 70L, 5M) 130, 22	–	–	+	+		Normal	MAS, I, ME	Not tolerating medication; problems with headache, nausea, vomiting, dizziness
60 y, M§	H	0	410 (8N, 91L, 1M) ND, ND	285 (45N, 50L, 5M) 191, 56	–	–	+	–		Normal	MAS	Home with change in mentation
66 y, M§	W	0	165 (13N, 87M) 357, 54	373 (28N, 72M) 373, 65	U	U	+	+		ND	MAS, I, BE, ME	Died
67 y, F§	B	5	320 (30N, 64L, 6M) 162, 128		–	–	+	–	Sputum AFB culture –, AFB smear –	Normal	ME	Improved mental status, transferred to nursing facility
72 y, M	A	5	106 (8N, 88L, 4M) 278, 22	130 (35N, 54L, 11M) 222, 18	–	–	+	–	Sputum AFB culture +, AFB smear –	Interstitial disease not considered typical for TB	I	Home in good condition
75 y, M	W	2	105 (37N, 44L) 187, 36	156 (74N, 22L) 223, 28	+ (1997)	–	+	–		Small calcified granuloma in the right mid lung and hilum	Normal	Stable, rehabilitation for strength training
77 y, F	W	5	178 (15N, 83L, 2M) 160, 40	110 (26N, 66L, 8M) 378, 12	+ (1974)	–	+	+		Normal	Normal	Died

*LP, lumbar puncture; CSF, cerebrospinal fluid; TST, tuberculin skin test; AFB, acid-fast bacilli; TB, tuberculosis (*M. tuberculosis*); CXR, chest radiograph; MRI, magmetic resonance imaging; A, Asian; ND, not done; L, lymphocyte; M, monocyte; +, positive; –, negative; MAS, multiple sites of abnormal signal; I, ischemia-infarct; H, hydrocephalus; BE, basilar enhancement; H, Hispanic; N, neutrophjl; U, unknown; E, eospinophil; ME, meningeal enhancement; G tube, gastrostomy tube; VP, ventriculoperitoneal; W, white; B, black.

†LP no. 1 CSF values, median (range): leukocytes 201 cells/mm^3 (42–2,845 cells/mm^3), protein 174 mg/dL (66–357 mg/dL), glucose 35 mg/dL (8–132 mg/dL), glucose excluding patients with diabetes 27 mg/dL (8–52 mg/dL).

‡LP no. 2 CSF values, median (range): leukocytes 285 cells/mm^3 (6–1,030 cells/mm^3), protein 196 mg/dL (87–381 mg/dL), glucose 28 mg/dL (5–88 mg/dL), glucose excluding patients with diabetes 22 mg/dL (12–63 mg/dL).

§Patient with diabetes.
